# ApoB-100 Lipoprotein Complex Formation with Intima Proteoglycans as a Cause of Atherosclerosis and Its Possible Ex Vivo Evaluation as a Disease Biomarker

**DOI:** 10.3390/jcdd5030036

**Published:** 2018-07-01

**Authors:** Eva Hurt-Camejo, Germán Camejo

**Affiliations:** 1 Division of Clinical Chemistry, Department of Laboratory Medicine, Karolinska Institutet, 141 86 Stockholm, Sweden; eva.hurt-camejo@astrazeneca.com; 2 Translational Sciences, Cardiovascular, Renal and Metabolism, IMED Biotech Unit, AstraZeneca, 431 83 Gothenburg, Sweden

**Keywords:** apoB-100 lipoproteins, proteoglycans, interaction, atherosclerosis

## Abstract

Experimental and clinical data indicate that the initiation and progress of atherosclerosis and its clinical manifestations are first caused by circulating apoB-100 lipoproteins that enter and are retained in the arterial intima. Extracellular sulfated proteoglycans (PGs) of the intima are the retention agents. The PGs also initiate physical and biochemical lipoprotein degradation with the production of bioactive, lipid products that trigger an inflammatory response that leads to atherosclerosis. There are many simple methods for measuring abnormalities of circulating lipoproteins and their relation to atherosclerotic cardiovascular disease (ACVD). However, limited research aims to evaluate procedures that could report quantitatively about the contribution of the interaction of apoB-100 lipoprotein-arterial intima PGs to clinical manifestation of ACVD. In the present review we discuss observations indicating that simple ex vivo evaluation of the affinity of apoB-100 lipoproteins for arterial PGs and glycosaminoglycans (GAGs) can give an indication of its association with clinical manifestations of atherosclerosis. In addition, we discuss molecular and cellular aspects of the apoB-100 lipoproteins association with arterial PGs that are related to atherogenesis and that support the experimental framework behind the current “Response-to-Retention” hypothesis of atherosclerosis.

## 1. Introduction

**Figure 1:**
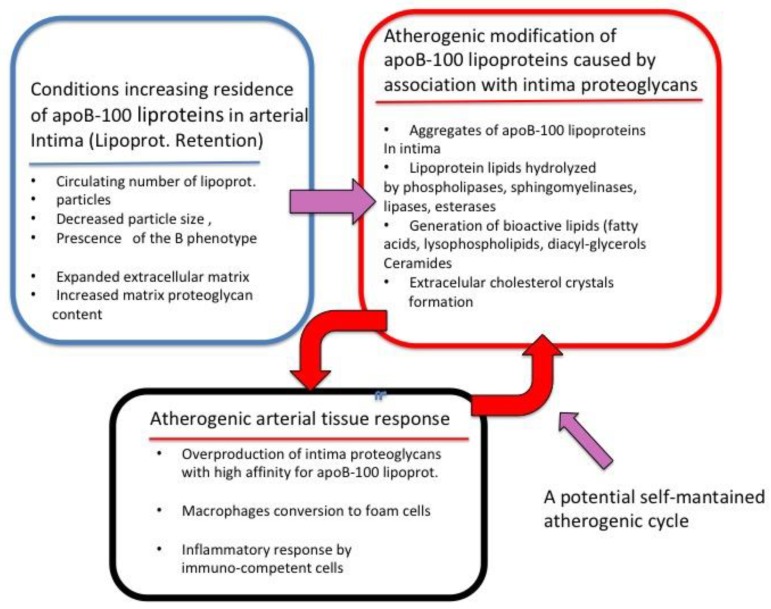
Summary of the main processes that supported the Response-to-Retention hypothesis of atherosclerosis.

The deposition of cholesterol-containing apoB-100 lipoproteins in the extracellular arterial intima is a critical initial step in the development of atherosclerotic lesions. A recent consensus document reviewed the evidence that connects the levels of circulating apoB-100 lipoproteins, and their interaction with the intima and the clinical events caused by atherosclerosis [1]. Mogen Faber, in pioneering studies, already suggested the possible mechanisms that linked plasma lipoproteins with atherosclerosis in humans using histochemical observations in 1949 [2]. Faber proposed that the cholesterol in human atherosclerotic lesions originated from circulating plasma lipoproteins. Furthermore, he found that the extracellular lipoprotein cholesterol deposits in the arterial intima-media were associated with extracellular sulfated polysaccharides [2]. Per-Henrik Iverious, in 1972, showed that the molecules that bind low density lipoproteins (LDL) connective tissue, including the arterial intima, were the sulfated polysaccharides (glycosaminoglycans, GAGs) of the PGs and he characterized the ionic nature of the association [3]. The biochemical nature of the lipoproteins-GAGs complex in human atherosclerotic lesion was firmly established by Srinivasan and collaborators in 1972 [4]. These studies have been subsequently extended and, the biochemical and molecular details of the processes generated by the interaction of apoB-100 lipoproteins and the intima PGs have been fitted into a coherent pathogenic sequence that is currently known as the “Response-to-Retention” hypothesis of atherosclerosis. Williams and Borén recently published an in-depth review of the experimental support for this concept [5,6,7]. A short version of the hypothesis can be described as follows: the “retention” part is related to the entrapment of apoB-100 plasma lipoproteins in the arterial intima by PGs of the extracellular matrix. Once retained, the cholesterol-rich apoB-100 lipoproteins coalesce as large lipid-protein aggregates that are partially degraded by enzymatic and oxidative pathways. Several of the byproducts of the retained apoB-100 lipoproteins are pro-inflammatory and can act on macrophages, smooth muscle cells and other immune competent cells triggering a complex inflammatory process that evolves into atherosclerotic plaques. This is the “response” part of the hypothesis [1,5,6,7,8]. Being the interaction of apoB-100 lipoproteins with arterial intima PGs such a central biochemical and pathogenic processes it seemed important to explore whether the basic mechanism can be used as an additional in vitro marker of the apoB-100 lipoproteins atherogenicity. In the present review, we summarized data suggesting that measurements of the affinity of apoB-100 lipoproteins for arterial PGs could be a biomarker of the atherogenic response. In Figure 1 below, we summarize the main processes behind the Response-to-Retention hypothesis, which is discussed in this review.

## 2. Ex Vivo Evaluation of the ApoB-100 Lipoprotein Proteoglycan Interaction and Its Possible Relation with ACVD

In this section we discuss the results of studies aimed to evaluate whether the affinity of LDL in plasma or serum for arterial PGs were associated with clinical manifestations of the risk for ACVD. Our laboratory found that soluble extracts of dissected human aortic intimas, obtained from young accident victims, contained a macromolecular component that formed an insoluble complex with LDL at near physiological conditions. The specific LDL precipitation by the arterial macromolecule also occurred when human plasma or serum were used. The arterial macromolecule was found to be versican, the most common chondroitin sulfate-rich PG of the human intima [9]. Using purified versican solutions, a standard procedure was developed in which the amount of complex of serum LDL with the arterial versican was measured ex vivo. This simple procedure can be used to compare the amount of complex formation with isolated LDL or with that LDL present in human serum or plasma samples. The results can be expressed as micrograms of LDL cholesterol (LDL-C) precipitated. The ionic conditions selected for this ex vivo measurement were based on studies of Berenfeld and collaborators, which established the required ionic composition of buffers needed for specific LDL precipitation with biological and synthetic sulfated polysaccharides [10]. We confirmed that, in the conditions of pH and with the buffer composition selected, more than 95% of the cholesterol precipitated by the versican was present as LDL (density range 1.019–1063 g/mL) when examined by density gradient ultracentrifugation, after re-solubilizing the LDL-PG complex. In a first study with human sera we evaluated the LDL-PG insoluble complex formation, measured as µg of insolubilized LDL-C, using 50 µL of serum or plasma added to 1 mL of the PG solution containing 10 µg PGs as hexuronate. The subjects were 291 adult males with no history of myocardial infarct and that had not taken lipid-lowering drugs. The subjects were classified as 214 apparent healthy and 77 probably ischemic using standardized exercise electrocardiography. It was found that the serum from the apparent ischemic subjects showed a higher prevalence of high values for LDL-PG complex formation (14–16 µg LDL-C) than the non-ischemic (6-8 µg LDL-C) [11]. 

In a following study, and in order to discard the possibility that the differences measured in the amount of LDL-PG complex formation were just the product of differences in LDL serum content, Lindén et al. compared the ex vivo LDL-PG affinity in myocardial young infarct patients with that of apparently healthy controls that were matched for age, sex, and levels of serum cholesterol, triglycerides, LDL-C, apoB and HDL-cholesterol [12]. In these well-characterized groups, the sera from myocardial infarct patients showed significantly higher values of precipitated µg of LDL-C (23.7 ± 5.3 vs. 15.6 ± 4.4, *p* < 0.0001). These differences remained highly significant when expressed as percentage of the cholesterol that was present in the serum aliquots which were added to the PG solution. Furthermore, LDL-PG complex formation appeared as an independent contributor following multiple regression analysis that, together with serum triglycerides, was able to discriminate patients and controls. There were other important observations in the study by Linden et al. [12]. They showed that freezing at –80 °C and thawing had minor effects on the LDL-PG precipitation measured, and that the analysis had a coefficient of variation of approximately 15%. Finally, and importantly, using density gradient ultracentrifuge analysis, the authors also confirmed that with the used protocol in the LDL-PG complex, once dissolved, more than 90% of the cholesterol was associated with lipoproteins with the density range of LDL, 1.019–1.063 g/mL. The gradient profiles showed no HDL in the dissolved pellets and less than 5% of the precipitated cholesterol was associated with the density of VLDL (<1.019 g/mL). 

Analysis of the LDL-PG complex formation has been applied to sera from patients with high cardiovascular risk (obese, hypertensive, with high triglycerides and hypercholesterolemia) that were subjected to multifactorial treatment [13]. The randomized patients (61 in the intervention group and 51 in the usual care group) were evaluated after 3 years of treatment compliance. The LDL-PG test, expressed as a percentage of added serum cholesterol, was −4.9% in the intervention group compared with the usual care group (*p* < 0.05). In a subsequent study, the effect of lipid lowering drugs on the LDL-PG association was analyzed by Wiklund et al. on moderate hypercholesterolemic patients [14]. The patients were randomized to pravastatin (40 mg), gemfibrozil (600 mg b.i.d), gemfibrozil and pravastatin (same doses) and placebo groups for 12 weeks. The drugs treatments showed the expected significant effects on serum triglycerides, total cholesterol, LDL-C and apoB and there were no changes in the placebo group. Differences between before and after treatments in measurements of the serum LDL-PG precipitation were very significant when expressed as absolute values or as percentage of serum cholesterol, of apoB or LDL-cholesterol added. These results also indicated that the LDL-PG precipitation test measured changes in affinity of LDL for the arterial PG and not only differences in LDL levels. A general observation in some of the previous studies was that the serum LDL of subjects with type 2 diabetes (T2D) or with insulin resistance produced more aggregates with arterial versican PG than subjects without these conditions. Garces et al. [15] explored this observation in a study in which LDL-PG affinity was measured in subjects with obesity but not type 2 diabetes (T2D), in subjects with obesity with TD2, in subjects with T2D but no diabetes and in apparently healthy controls with no obesity or T2D The results showed that obesity, with or without T2D, was associated with significantly higher values of plasma LDL-PG precipitation (18 µg LDL-C precipitated/mg apoB added) compared to 11.7 µg of LDL-C precipitated/mg apoB added in subjects with no obesity. Interestingly, the elevated levels of LDL-C-PG complex were strongly associated with levels of serum phospholipase A_2_ and with high prevalence of smaller LDL particles. We have discussed this aspect below. The summarized clinical studies indicated that increased LDL affinity for arterial versican was associated with increased clinical markers of atherosclerosis and ACVD risk. 

Two solid phase procedures for measuring apoB-100 lipoproteins binding to PGs have been described that use a microtiter plate format [16,17]. In these procedures, the PGs were attached to the plates and the solutions of isolated lipoproteins, or in plasma, were incubated in a buffer similar to the one used in the liquid phase procedure we described. The plates were washed of non-bound lipoproteins and the PG-retained apoB-100 lipoproteins were determined with an immune-assay, or the cholesterol in the retained apoB-100 lipoproteins measured. These solid phase methods could be easily automatized, thus increasing their application in the analysis of many samples. 

## 3. ApoB-100 Lipoprotein Entry and Its Proteoglycan-Mediated Retention in the Arterial Intima

The steady state concentrations of soluble lipoproteins in the arterial intima depend of their rate of entry and exit [1]. The endothelial barrier appears to exclude particles with diameter above 70 nm in diameter, such as very large VLDL and chylomicrons. VLDL remnants, LDL and HDL, which can cross the endothelium, are present in the intima in amounts that are inversely proportional to their size and directly proportional to their concentration in human serum and plasma [1,6,7,18,19] Because of its higher plasma concentration LDL exists at the highest concentration in the extracellular arterial intima, probably at 20 times the value for VLDL remnants [1,19]. There is a linear correlation between the levels of circulating cholesterol-rich apoB-100 lipoproteins and the content of immune-detectable LDL in early atherosclerosis in humans, as shown by Smith and Slater [18]. Because of this, the strong association between plasma apoB-100 lipoproteins and the clinical manifestations of atherosclerosis is to be expected [1]. In human early coronary atherosclerosis, lipoprotein deposition in proteoglycan-rich regions of the intima occurs before macrophage infiltration [20]. These results support the importance of interactions between apoB-100, cholesterol-rich, lipoproteins intima PGs and early atherogenesis. 

Retention of lipoprotein particles in the intima occurs first by association of specific basic segments of the apoB-100 protein, which are rich in lysine and arginine, on the particle surface. These charged amino-acid sequences can form soluble and insoluble complexes with the sulfate groups of the glycosaminoglycan (GAG) moiety of PGs. Our laboratory used frontal affinity chromatography and competition experiments with synthetic peptides of the apoB-100 to identify the main GAG-binding sequences of the protein. Two sequences: 3145–3157 (-Ser-Val-**Lys**-Ala-Gln-Trp-**Lys**-**Lys**-Asn-His-**Arg**-His) and 3359–3367 (-**Arg**-Leu-Thr-**Arg**-**Lys**-**Arg**-Gly-Leu-**Lys**-) (Bold indicate positive charged amino acids) provided the affinity for versican, the main PG of the human intima. Both sequences hade 5 positive charged residues (Lysine and Arginine) and they had similar affinities for versican. The affinity was 6–7 µm/L when expressed as inhibition constant (IC50) [6,21]. Borén and collaborators, in a series of important experiments, used mutagenesis in the human apoB-100 gene within the coding region for the 3359–3367 PG-binding sequence. They demonstrated, in a hypercholesterolemic mouse model, how important this basic segment was for the in vivo PG-mediated LDL retention and consequently for early atherosclerosis progress [7,16].

LDL in plasma can be associated with other apolipoproteins like apoE, apoAI and apoC-III [22], which can modulate its affinity for PGs. Davidson et al. [22] showed, with a proteomic approach, that subjects with peripheral atherosclerosis and type 2 diabetes (T2D), that also have LDL with a high prevalence of small, dense particles, contained elevated levels of all apoC-III isoforms, when compared with the LDL of healthy controls. Furthermore, the apoC-III content in their small, dense LDL was positively correlated with rates of LDL binding to PGs [22]. In related experiments, Olin-Lewis et al. found that the affinity of LDL subclasses for the PG biglycan increased with the decreasing diameter of the particles and that this trend was associated with the apoC-III content [23]. Furthermore, Hiukka et al. [24] examined the PG-binding of LDL rich in apoC-III from patients with T2D that frequently had high levels of small, dense LDL. These lipoproteins also showed an increased binding for the small PG biglycan. It is not clear why augmented apoC-III content increased LDL-PG binding. It may have been that this occurred because smaller LDL particles had more ApoC-III copies on their surface and that their intrinsic, size-related affinity was the real cause. 

There are other proteins that can associate with apoB-100 lipoproteins in circulation, or once they have entered the sub-endothelial space. Several of them appear to increase the affinity of the apoB-100 lipoproteins for intima PGs, thus creating a ternary complex that can increase the lipoproteins’ retention. The best documented are lipoprotein lipase, phospholipase A_2_ and sphingomyelinase [25,26,27]. This probably occurs because the enzymes have two surface recognition regions, one for the intima PGs and other for the apoB-100 lipoproteins. Or possibly because the enzymatic hydrolysis of lipoprotein lipids causes lipoprotein aggregation [25,26,27]. In relation to this, the formation of apoB-100 lipoprotein aggregates, induced by PGs, is strongly affected by the pH of the media, reaching a maximum when the pH gets closer to the isoelectric point (pI) of the lipoprotein (pI approx. 5.85). This has important implications for the interactions taken place in the intima in early atherogenesis where an acidic environment has been detected [28]. In relation to this, our laboratory found that LDL subclasses with the higher pI, (more basic) were also the smaller, denser fractions that had more affinity for arterial PGs [29]. Thus small, dense LDL particles may have been more prone to aggregation. Öörni et al. have reviewed this topic in depth [28].

Recently, an original set of experiments has added experimental support to the important contribution of the sulfated GAGs of PGs to the progress of atherosclerosis mediated by accumulation of apoB-100 lipoproteins. Vazques and collaborators [30] developed a chimeric mouse/human monoclonal antibody (mAb), chP3R99-LALA that was directed against the chondroitin sulfate GAG chains of PGs. This mAb was used with a vaccination protocol roach in cholesterol-fed apoE^−/−^ deficient mice. The animals receiving the mAb showed an arrest of lesion development with a reduction in macrophage infiltration and a decrease in markers of free-radical mediated oxidation. The results, discussed above, left little doubt about the critical participation of specific basic peptide sequences in the apoB-100 lipoproteins and the sulfated GAGs of PGs during lipoprotein retention in the intima. 

## 4. Atherogenic Structural and Biochemical ApoB-100 Lipoprotein Alterations Caused by PG Retention

The transition from retention in the intima to lipoprotein-mediated alterations of cellular constituents of the intima, leading to the “response” phase, is probably very fast. These rapid alterations are initiated by the physico-chemical interaction of apoB-100 lipoproteins with the extracellular and peri-cellular sulfated PGs. In this situation it is difficult to separate the processes contributing to the “response” and those contributing to the “retention”. Low angle X-ray diffraction and scanning calorimetry experiments have indicated that, once bound to the GAGs of arterial versican, the apoB-100 lipids in the particles in the complex become rapidly disorganized and can form large soluble and insoluble fused lipoprotein aggregates [31]. Furthermore, the surface exposure of the apoB-100 basic segments at the particles surface is increased and the protein becomes more susceptible to proteolysis [32]. Large aggregates of apoB-100 lipoproteins have been detected with high-resolution electron microcopy in the arterial intima of rabbits after a human LDL bolus infusion [33]. This indicated that the association of LDL with the extracellular matrix took place very rapidly, alongside the formation of large aggregates, and before macrophages recruitment [20,33]. The biochemical changes in the apoB-100 particles took place after apoB-100 lipoproteins association with the arterial matrix had been documented in human intima-media segments with different types of atherosclerotic lesions. Most studies have shown that lipid and protein moieties of the extractable apoB-lipoproteins from the intima suffered rapid physical and hydrolytic modifications, see review by Hoff and Hoppe [34]. Our laboratory showed that lipoproteins that could be extracted from human lesion and purified by affinity chromatography with anti-apoB-100 antibodies chromatography contained particles with the size of LDL and large apoB-100 aggregates that also contained GAGs. Analysis of the fatty acids of phosphatidyl choline, cholesteryl esters and triglycerides from the extracted arterial lipoprotein showed approximately 50% depletion of linoleic acid in all the phospholipids and cholesteryl esters. This suggested that apoB-100 lipoproteins, with still immuno-reacting apoB, were associated with PGs and had been targets of different phospholipases and cholesteryl esterases and also for possible oxidative processes of unsaturated fatty acids [35]. Lipolytic hydrolysis of phospholipids, and cholesteryl esters from retained apoB-100 lipoproteins have generated fatty acids that can generate free-radicals, thus producing further pro-inflammatory products. In addition, in situ production of lysophospholipids and diacyl-glycerol has also mediated the activation of inflammatory cascades in macrophages, endothelial cells and muscle cells [36]. 

## 5. Cellular Consequences of the Association of ApoB-100 Lipoproteins with Intima Proteoglycans

As already discussed, interaction of LDL with the human arterial PG versican has altered the physico-chemical structure of the particles [6] without necessarily inducing aggregation. One of the most significant effects of such modification is how human macrophages increased the binding and uptake of the soluble PG-modified LDL. Furthermore, the PG-modified LDL caused appreciable intracellular accumulation of free cholesterol, cholesteryl esters, triglycerides and phospholipids, resulting in appreciable formation of intracellular lipid vacuoles [36]. This increase in lipid cell content was driven by the uptake of LDL through the apoB/E receptor and by endogenous lipid synthesis by the macrophages. We observed that LDL preparations from different blood donors were internalized at different rates in the macrophage experiments. Thus, we explored whether different LDL subclasses with dissimilar affinity for PGs could have had unique structural properties, and furthermore whether the macrophages internalized the subclasses at different rates after their association with the human arterial versican [29]. These experiments were prompted by results which showed that LDL subspecies with different density and sizes have specific lipid composition and different conformations of the apoB-100 [37]. We found that that LDL subclasses, selected by their increasing affinity for human arterial versican, were associated with decreased diameter and volume due to an augmented ratio of surface components to core lipids (cholesteryl esters). As mentioned, the smaller LDL fractions were associated with increasing isoelectric point (more basic). In addition, the smaller LDL subclasses were internalized more efficiently by the human macrophages and were found more susceptible to free-radical mediated oxidation [6,29]. All these properties of the LDL subclasses with high affinity for arterial PG can be considered atherogenic [6]. The increased atherogenicity of small, dense LDL particles compared with large, less dense ones, has been ascribed to easier entry into the intima, to a higher affinity for intima PGs and to higher susceptibility to hydrolytic and oxidative modification of the small, dense LDL [38,39,40]. We hypothesized that all these potentially atherogenic properties were related to increased exposure of the PG-binding, polar segments of apoB-100 on the particle surface [6,36]. More recently, Flood et al. [38], using recombinant protein experiments, showed that conformational changes in the apoB-100 sequences 3145–3157 (PG-binding site A) and 3359–3967 (PG binding site B) in small, dense LDL acted cooperatively to increase its affinity for intimal PGs. LDL treatment with sPLA_2_ that reduced the surface components of the particle also increased the affinity for PGs. Furthermore, extended action of phospholipases on circulating apoB-100 lipoproteins was a probable generator of small, dense LDL with higher PG affinity [27,36]. Therefore, the B phenotype with a high prevalence of small, dense LDL and a high frequency of insulin resistance and T2D may have been especially atherogenic for the particles’ increased affinity for PGs and subsequent biochemical susceptibility to alterations. 

## 6. Alterations of the Arterial Intima Extracellular Matrix that May Contribute to Increased Retention of ApoB-100 Lipoproteins

One of the characteristics of initial atherosclerotic lesions is the intimal thickening. These intimal regions, rich in PGs, are the preferred site for extracellular apoB-100 lipoprotein retention and lipid accumulation [7,20,41]. The cells generating the extracellular matrix of these regions are smooth muscle cells, and possibly macrophages [20,41]. Biochemical and cell studies showed that phospholipase A_2_ (PLA_2_), which can enter these PG-rich regions when bound to lipoproteins or when being secreted by intimal cells, form tight associations with PGs, and in this form increase its activity towards the phospholipids of PG-attached lipoproteins [27,40]. This situation can further potentiate the generation of pro-inflammatory active lipids as non-esterified fatty acids, oxidized fatty acids and lysophospholipids and increase the affinity of apoB-100 lipoproteins for the intima PGs [27,40]. Increased exposure of arterial smooth muscle cells to non-esterified fatty acids, as may occur in insulin resistance or T2D, may have further atherogenic consequences since it was found that this can augment the secretion of extracellular matrix proteoglycans with increased affinity for apoB-100 lipoproteins [42,43]. Because of this, the PG-mediated “retention” of apoB-100 lipoproteins can initiate a self-perpetuating process resulting in an enhanced atherogenic “response” of augmented matrix production with more capacity to retain apoB-100 lipoproteins [43]. 

There are other conditions that can cause matrix production with an increased capacity to retain apoB-100 lipoproteins mediated by PGs. Recently, Kijani et al. found that vascular interventions in the common carotid of mice, resulting in intimal hyperplasia, induced deposition of apoB-100 lipoproteins and rapid atherosclerosis [44]. Further confirmation of the importance of the extracellular matrix proteoglycan structure on retention of apoB lipoproteins in atherogenesis was recently reported by Bach Steffensen and collaborators [45]. The authors used fluorescent-labeled LDL and HDL to first locate the natural sites of lipoprotein retention in mouse arteries. These sites were identified as the inner aortic arch, nearby branches and sites of disturbed laminar flow. On the other hand, straight artery segments with undisturbed laminar flow showed minimal labeled protein retention. The authors then anatomically induced disturbed laminar flow in the straight segments of arteries. This intervention caused hyperplasia and early plaques in six weeks. These sites also became locations for massive retention of the labeled lipoproteins. Interestingly, in these sites, increased expression of genes associated with the smooth muscle cells phenotype and augmented expression of genes for the core proteins of lipoprotein-retaining PGs and for enzymes responsible for GAGs biosynthesis [44] was observed. These results again pointed out the connection between apoB-100 lipoprotein retention and a cellular response that further increased an atherogenic response. 

## 7. Conclusions

Extensive experimental and clinical data consistently supported the premises on which the “Response-to-Retention” hypothesis of atherosclerosis is based. Moreover, the cellular and biochemical bases of the hypothesis provided the rationale for further developments in current and new anti-atherosclerotic treatments centered in the correction of dyslipidemias. Specially, those contributing to the deposition of cholesterol-rich apoB-100 lipoproteins in the arterial intima-media, which are causal of atherosclerotic cardiovascular disease. In addition, the data discussed about how the structure and amount of the PGs of lesion-prone sites also control apoB-100 lipoproteins retention suggested that blocking hyperplasia or modulating PGs expression may be a novel therapeutic area. The discussed preliminary studies about the possibility of identifying biomarkers of the molecular interactions between atherogenic apoB-lipoproteins and the arterial intima were encouraging. We believe that it is important to further evaluate these non-invasive simple markers that could be used for measuring the predisposition of lipoproteins to be retained in the intima. Specially, there is a need for prospective studies aiming to evaluate the predictive value of ex vivo measurements of apoB-100 lipoproteins affinity for PGs as a marker of the central processes behind ACVD.
